# A *De novo* Transcriptomic Approach to Identify Flavonoids and Anthocyanins “Switch-Off” in Olive (*Olea europaea* L.) Drupes at Different Stages of Maturation

**DOI:** 10.3389/fpls.2015.01246

**Published:** 2016-01-19

**Authors:** Domenico L. Iaria, Adriana Chiappetta, Innocenzo Muzzalupo

**Affiliations:** ^1^Consiglio per la Ricerca in Agricoltura e l'Analisi dell'Economia Agraria, Centro di Ricerca per l'Olivicoltura e l'Industria OleariaCosenza, Italy; ^2^Dipartimento di Biologia, Ecologia e Scienze della Terra, Università della CalabriaCosenza, Italy; ^3^Dipartimento di Farmacia, Scienze della Salute e della Nutrizione, Università della CalabriaCosenza, Italy

**Keywords:** *Olea europaea*, flavonoid and anthocyanin pathway, RNA-seq, *de novo* assembly, gene expression

## Abstract

**Highlights**

- A *de novo* transcriptome reconstruction of olive drupes was performed in two genotypes
- Gene expression was monitored during drupe development in two olive cultivars
- Transcripts involved in flavonoid and anthocyanin pathways were analyzed in Cassanese and Leucocarpa cultivars
- Both cultivar and developmental stage impact gene expression in *Olea europaea* fruits.

During ripening, the fruits of the olive tree (*Olea europaea* L.) undergo a progressive chromatic change characterized by the formation of a red-brown “spot” which gradually extends on the epidermis and in the innermost part of the mesocarp. This event finds an exception in the Leucocarpa cultivar, in which we observe a destabilized equilibrium between the metabolisms of chlorophyll and other pigments, particularly the anthocyanins whose switch-off during maturation promotes the white coloration of fruits. Despite its importance, genomic information on the olive tree is still lacking. Different RNA-seq libraries were generated from drupes of “Leucocarpa” and “Cassanese” olive genotypes, sampled at 100 and 130 days after flowering (DAF), and were used in order to identify transcripts involved in the main phenotypic changes of fruits during maturation and their corresponding expression patterns. A total of 103,359 transcripts were obtained and 3792 and 3064 were differentially expressed in “Leucocarpa” and “Cassanese” genotypes, respectively, during 100–130 DAF transition. Among them flavonoid and anthocyanin related transcripts such as phenylalanine ammonia lyase (PAL), cinnamate 4-hydroxylase (C4H), 4-coumarate-CoA ligase (4CL), chalcone synthase (CHS), chalcone isomerase (CHI), flavanone 3-hydroxylase (F3H), flavonol 3′-hydrogenase (F3′H), flavonol 3′5 ′-hydrogenase (F3′5′H), flavonol synthase (FLS), dihydroflavonol 4-reductase (DFR), anthocyanidin synthase (ANS), UDP-glucose:anthocianidin: flavonoid glucosyltransferase (UFGT) were identified. These results contribute to reducing the current gap in information regarding metabolic processes, including those linked to fruit pigmentation in the olive.

## Introduction

The olive tree (*Olea europaea* L. subsp. *europaea* var. *europaea*) is one of the most important and widespread fruit trees in the Mediterranean area. It belongs to the *Oleaceae* family, which includes 600 species within 25 genera. It is widely distributed on all continents, from temperate areas in the north to sub-tropical regions and from low to high altitudes. Native to Mediterranean regions, *Olea europaea* is the only species within the genus *Olea* that produces edible fruits (Green and Wickens, 1989; Wallander and Albert, 2000; Green, 2002; FAOSTAT, 2008^1^). The quality of its products, olive oil and table olives, is highly dependent on the agronomic and organoleptic characteristics of its drupes. These characteristics vary in relation to the genetic traits, varieties, the stage of ripeness, as well as in relation to the different susceptibility to environmental growth conditions (Loumou and Giourga, 2003; Conde et al., 2008).

The genuineness of olive oil is important within the “Mediterranean diet.” Several research and epidemiological studies link healthy aspects of its components; in particular, olive oil is known to exert protective effects against vascular disease and the onset of cancer (Vauzour et al., 2010). These features are correlated to the high percentage of monounsaturated fats as well as to the high content of antioxidant compounds such as phenols and tocopherols, which, together with other components, characterize the nutraceutical profile of olive products (Pérez-Jiménez et al., 2007; Bruno et al., 2009; Muzzalupo et al., 2011). Phenolic compounds represent a complex mixture in olive derived products responsible for the anti-atherogenic and anti-cancerogenic effects, and for antioxidant properties (Hashim et al., 2008; Llorente-Cortes et al., 2010; Martinelli and Tonutti, 2012). Despite the importance and uniqueness of olive products, the long juvenile developmental phase and its intrinsic self-incompatibility mechanisms slow down current olive breeding programs, which are still very long. Although the current breeding strategies can now benefit from the availability of new polymorphic genetic markers, characterization of the olive germplasm is still far from complete (Baldoni et al., 2009; Muzzalupo, 2012; Muzzalupo et al., 2014).

Therefore, it is of prime importance to focus research programs toward innovative improvement strategies to support conventional programs. In particular, a wider characterization of genes related to plant product quality and to adaptive mechanisms, could provide new information and tools to support both Marker Aided Selection (MAS) strategies and biotechnological approaches. This would aid the development of new growing techniques to increase productivity and quality of this unique species.

Anthocyanins are the most widely distributed group of pigments in plants. They are synthesized via the phenylpropanoid pathway and are mainly responsible for the mauve, red, blue, and purple colors in flowers, fruits, leaves, seeds, and other organs in most flowering plants. As one of the most ubiquitous class of flavonoids, anthocyanins possess a multitude of biological roles, including protection against solar exposure and ultraviolet radiation, free radical scavenging and anti-oxidative capacity, defense against many different pathogens, and attraction of predators for seed dispersal. Anthocyanins also play a role in consumer preference for flower and fruit quality, potential food health properties, and related horticultural attributes. As a result, classical breeding, as well as transgene technologies, have been used to enhance or create novel colors in ornamental and food crops (Chalker-Scott, 1999; Schaefer et al., 2004; Takahama, 2004; Stommel et al., 2009).

The enzymes involved in the anthocyanin biosynthetic pathway are well characterized. Many of the genes encoding these enzymes have been cloned and share high sequence similarity across species and exhibit tissue- or development-specific expression. Chalcone synthase (CHS) is the first enzymatic step of the biosynthetic pathway (Coe et al., 1981; Dooner, 1983; Koes et al., 1989, 2005). Subsequently chalcone isomerase (CHI) catalyzes the isomerization of chalcone to naringenin (van Tunen et al., 1988, 1989; Grotewold and Peterson, 1994; Griesbach and Beck, 2005). Flavanone 3-hydroxylase (F3H) converts naringen into dihydrokaempferol, which is converted to anthocyanins by the action of three enzymes. Dihydroflavonol is first converted to a colorless leucoanthocyanidin by dihydroflavonol 4-reductase (DFR). Leucoanthocyanidins are subsequently converted to colored anthocyanidins by anthocyanidin synthase (ANS) finally, the UDP-glucose-flavonoid 3-O-glucosyltransferase (UFGT) creates the anthocyanin-3-glucoside. Within the path, the CHS is the first and key regulatory enzyme of flavonoid biosynthesis and the DFR is the first committed enzyme of anthocyanin biosynthesis in the flavonoid pathway (Holton and Cornish, 1995; Ramsay and Glover, 2005; Martinelli and Tonutti, 2012).

Despite having been recently studied in different olive cultivars (Alagna et al., 2009; Galla et al., 2009; Martinelli and Tonutti, 2012) the molecular mechanisms involved in the regulation of biosynthesis are still unknown.

Tissue- or developmental-specific expression exhibited by anthocyanin structural genes is controlled by a set of regulatory genes. It is known that MYB, bHLH MYC, and WD40 repeat proteins, interacting together to form a regulatory complex that controls anthocyanin structural genes at the transcriptional level (Dixon et al., 2005; Ramsay and Glover, 2005; He et al., 2008; Tian et al., 2008; Alagna et al., 2009; Galla et al., 2009; Stommel et al., 2009; Martinelli and Tonutti, 2012; Ravaglia et al., 2013; Chiappetta et al., 2015).

It has been suggested that a functional MYB-MYC-WD complex directly binds the cis-element of structural gene through MYB transcription factor, while R-like MYC might bind indirectly via a hypothetical R interaction protein (RIP) (Ramsay and Glover, 2005). R-like MYC is centered in the complex that interacts with a MYB factor with WD proteins on its sides. Together, they activate the entire set of anthocyanin biosynthesis genes (Stommel et al., 2009).

The aim of this work was to define the main transcriptomic profile differences during olive drupe development and to identify the transcripts involved in flavonoid and anthocyanin metabolism.

We have chosen to analyze the transcriptome profile at 100 and 130 days after flowering (DAF), through an Illumina RNA-seq approach, to identify the transcripts along flavonoids and anthocyanins biosynthetic pathways and to monitor their expression levels during ripening. A *de novo* transcriptome reconstruction of olive fruits was performed together with a full expression analysis between samples from “Leucocarpa,” an olive variety characterized by a switch-off in skin color at full ripeness, and “Cassanese,” used as control plant. Significant differences in flavonoid and anthocyanin transcript expression profiles emerged, both during fruit maturation and in relation to genotypes. Consequently, from the wide array of information obtained, our attention was focused on the identified candidate genes set, the expression of which was confirmed by quantitative PCR. In addition, the expression patterns of different MYB, MYC, and WDR transcriptional activators was compared to CHS, DFR, and ANS genes during fruit ripening (Matus et al., 2009; Ravaglia et al., 2013).

## Materials and methods

### Plant materials

Olive drupes, of *Olea europaea* L. Leucocarpa and Cassanese cv were used. Drupes were collected from 20-year-old plants, clonally propagated and belonging to the olive germplasm collection of the Agricultural Research Council—Olive Growing and Oil Industry Research Centre, CREA-OLI in Mirto-Crosia (Cosenza, Calabria, Italy). Olive trees were grown using the same field conditions and were located at latitude 39°37′04.57″N, longitude 16°45′42.00″E and altitude 8 m asl).

Fruit sampling was performed as previously described (Matus et al., 2009): for each cultivar, drupes (*n* = 30,) were randomly collected at 100 and 130 DAF (Figure S1). In order to minimize the effects related to asynchronous fruits maturation within the same tree, drupes with similar pigmentation were picked from all around the external parts of the tree canopy. Concerning drupe pigmentation, the epi-mesocarp tissues, was totally green in color at 100 DAF whereas at 130 DAF the pulp pigmentation was 50% brown in “Cassanese” and totally unpigmented in “Leucocarpa” drupes (Figure S1).

All samples were fixed in liquid nitrogen and stored at −80°C for both RNA-seq and qRT-PCR experiments.

### RNA-Seq library preparation and sequencing

In order to obtain a general overview of the transcripts and metabolic pathways involved in fruit maturation and to avoid cross contamination from non-homogeneous tissue separation, sample pooling strategy has been here used (Peng et al., 2003). Pooling reduces variability by minimizing individual variation and represents an alternative approach to biological replicates in experiments where the interest is not on the individual but rather on characteristics of the population (e.g., common changes in expression patterns; Karp and Lilley, 2007, 2009).

Total RNA was extracted from the epi-mesocarp tissues of drupes collected together, using the RNeasy Plant Mini kit (Qiagen) according to the manufacturer's instructions. Each RNA sample was subjected to DNase digestion (DNase I, Roche) to remove any DNA contamination and pooled equally, as previously described (Muzzalupo et al., 2012). RNA was quantified by the NanoDrop Spectrophotometer ND-2000 and quality was checked by electrophoresis (28S rRNA/18S rRNA ratios). Samples with a concentration of ≥400 ng/μl, OD260/280 = 1.8~2.2, RNA 28S:18S ≥ 1.0, and RNA Integrity Number (RIN) ≥ 7.0 were used for cDNA library preparation.

Standard RNA-seq library preparation and sequencing via Illumina HiSeq TM 2000 was carried out by Technology Services of the Institute of Applied Genomics (IGA, Udine, Italy). For each sample a single-end (SE) sequencing cDNA library was constructed with a fragment length range of 50 bp. Each library was created using two replicates, consisting of a separate pool of 30 homogeneous fruits.

### RNA-Seq data filter and *de novo* assembly by trinity

The raw Fastq “reads” (NCBI PDA/SRAaccession numbers: SRR1574719, SRR1574772, SRR1573503, SRR1574328, Table 1) were analyzed and filtered, respectively with FastQC and Fastx Toolkit softwares to obtain high quality *de novo* transcriptome sequence data. Each sequence set was filtered using the following criteria: (*i*) reads containing the sequencing adaptor were removed; (*ii*) reads with unknown nucleotides comprising more than 5% were removed; (*iii*) low-quality reads with ambiguous sequence “N” were trimmed and discarded.

**Table 1:** **Assembled transcripts for each sample**.

**Sample**	**Raw reads**	**Used reads**	**Assembled transcripts**	**Contig N50**	**Mapped reads**
Leucocarpa 100 DAF	28,700,100	23,687,921	22,959	754	84.07%
Leucocarpa 130 DAF	28,121,963	23,122,308	26,203	829	84.15%
Cassanese 100 DAF	28,550,901	23,394,526	22,709	767	83.82%
Cassanese 130 DAF	57,106,631	48,153,012	31,485	972	85.49%

Since the olive tree does not have a reference genome, the *de novo* assembly of the clean reads into transcripts was performed using the Trinity program (Grabherr et al., 2011; Haas et al., 2013), a useful method for the efficient and robust *de novo* reconstruction of transcriptomes from RNA-seq data (Ward et al., 2012; Gutierrez-Gonzalez et al., 2013; Liang et al., 2013; Liu et al., 2013; Pallavicini et al., 2013; Tulin et al., 2013).

Trinity was run via script using 128 GB of ram, 12 cpu thread and a minimum assembled contig length to report set to 300 bp.

Trinity sequentially combines Inchworm, Chrysalis and Butterfly modules to process large RNA-seq reads data, partitioning the sequence data into many individual de Bruijn graphs, representing transcriptional complexity at a given gene or locus (Grabherr et al., 2011; Haas et al., 2013).

### Analysis of transcript assembly

For non-model organisms, one metric for evaluating the transcript assembly quality is to examine the number of transcripts that appear to be full-length or nearly full-length if compared to a closely related organism to examine full-length coverage. In this context, a more general analysis was performed aligning the assembled transcripts against all known plant proteins determining the number of unique top matching proteins that are aligned in 70–100% range of its length by full-length transcript analysis (Haas et al., 2013). Therefore, a blastable database has been created to perform a local blastx search where only the single best matching Trinity transcript was outputted for each top matching entry.

To validate our de novo assembly read remapping has been realized using bowtie2 (Langmead and Salzberg, 2012); for each data set a bowtie2 index was created, and then the number of reads that map to our transcriptome have been counted.

### Abundance estimation and differentially expressed trinity transcripts

For abundance estimation of transcriptome assemblies RSEM software was used (Li and Dewey, 2011). RSEM is a package for estimating gene and isoform expression levels from RNA-seq data. The current version of RSEM, was bundled with the Trinity software package.

Moreover, Trinity currently supports the use of Bioconductor tools (edgeR and DESeq) to compute differential expression analysis in the assembled transcriptome (Anders and Huber, 2010; Robinson et al., 2010; Grabherr et al., 2011; Haas et al., 2013). In order to identify statistically significant differences in transcript expression between samples, the number of reads/transcripts, the depth of sequencing, the transcripts length (longer transcripts generate more fragment reads) and the expression level of the transcripts were considered. Expression values, normalized for each of these factors were measured in FPKM (fragments per feature kilo base per million reads mapped) (Trapnell et al., 2010; Robinson and Oshlack, 2010) and used to make a comparison across multiple samples and replicates. Trinity supports the use of TMM (trimmed mean of *M*-values) normalization (Lekanne Deprez et al., 2002; Dillies et al., 2012), to account for differences in the mass composition of the RNA-seq samples, which does not change the fragment count data, but provides a scaling parameter that yields an effective library size (total map able reads) for each sample. This effective library size is then used in the FPKM calculations.

### Quantitative PCR

Gene expression analysis was performed by quantitative real-time PCR on a 7500 fast real time PCR system (Applied Biosystems) with SYBR® Select Master Mix. The oligonucleotide primer sets (Table 1) used for qRT-PCR analysis were designed using Primer3 (http://primer3.ut.ee/).

Each primer pair (Supplementary data, Table S1) generated a single specific amplicon on the 3′-end of target sequence. PCR products were about 150–200 bp long and primer pair average efficiency ranged between 0.95 and 1.0. The housekeeping olive ELONGATION FACTOR 1 (EF1) gene (CAQ17046.1) was used to normalize the expression levels (Galla et al., 2009; Trapnell et al., 2010). Amplification reactions were prepared in a final volume of 20 μl according to the manufacturer's instructions.

All reactions were run in triplicate in 96-well reaction plates, and negative controls were set. The cycling parameters were as follows: one cycle at 95°C for 3 min to activate the Taq enzyme, followed by 40 cycles of denaturation at 95°C for 10 s and annealing-extension at 58°C for 30 s. To confirm the occurrence of a unique PCR product, the “melting curve” (Lekanne Deprez et al., 2002) was evaluated by an increase of 0.5°C every 10 s within a 60–95°C range and a unique “melting peak” in every reaction was observed. The comparisons of cycle threshold (CT) values were obtained analysing data with the 2^−ΔΔCT^ method (Livak and Schmittgen, 2001). The means of gene expression levels were calculated from two biological repeats, obtained from two independent experiments.

### Blast2GO

To assign gene ontology (GO) terms in our DE data sets, we used BLASTx 2.2.26+, BLOSUM62 similarity matrix, and Blast2GO database version August 2011 programs (Conesa et al., 2005; Morgulis, 2008). The definition of each GO term was determined by the GO Consortium: http://www.geneontology.org and can be found using the EMBL European Bioinformatics Institute QuickGO: http://www.ebi.ac.uk/QuickGO or the Gene Ontology Normal Usage Tracking System, GONUTS: http://gowiki.tamu.edu/wiki/index.php/Main_Page.

Pathway assignments were determined following the Kyoto Encyclopedia of Genes and Genomes pathway database (Kanehisa et al., 2008) using BLASTX with an *E*-value threshold of 1.0E-5.

MapMan (http://mapman.gabipd.org/) analysis was done using our DE transcripts rearranged as input experimental dataset. Using the Mercator web application we can assign MapMan “Bins” to DNA sequences (Thimm et al., 2004; Lohse et al., 2014). The output was used as a mapping file for data visualization in MapMan. The Mercator tool generates functional predictions by searching a variety of reference databases (BLAST-based, RPSBLAST based and InterProScan) and subsequently evaluating and compiling the search results for each input gene to propose a functional Bin.

## Results

### RNA-Seq library sequencing and de novo transcriptome assembly by trinity

Starting from four RNA-seq libraries, corresponding to two fruit developmental stages (100 and 130 DAF) of *Olea europaea* “Leucocarpa” and “Cassanese,” 147,789,544 raw reads were generated from 50 bp insert library. A total of 142,479,595 high-quality SE reads were identified and used for *Olea europaea* trascriptome assembly, through the Trinity software. Using the 25-mer in Trinity and a minimum assembled contig length set to 300 bp, we found 103,359 transcripts. The total used reads, the total assembled transcripts, N50 statistics for each sample and remapping results are indicated in Table 1.

A total of 93,623 likely coding sequences were extracted with the Transdecoder utility, to identify the longest ORF (Open Reading Frame) from the transcript assembly, reporting that ORF scored according to the Markov model. In all, 9597 of the olive transcripts had a BLAST hit with an *E*-value of less than 1e-20, and 19,708 of the extracted reference coding sequences are considered to be approximately “full length,” with the Trinity contigs aligning the matching UniProt reference transcript's length by more than 70%.

### Differential expression analysis

To estimate the differential gene expression between fruits of both considered cultivars at each developmental stage, a single assembly, based on combining all reads across all samples as inputs was generated. A single assembly was chosen to avoid difficulty in comparing the results across the different samples, due to differences in assembled transcript lengths and contiguity. Then, reads were aligned separately back to the single assembly, in order to identify the number of DE transcripts with a False Discovery Rate (FDR) value of at most 0.001 and at least four-fold difference in expression values according to the Trinity protocol.

For this purpose, it was possible to identify the DE transcripts sets of each cultivar, during the 100–130 DAF transition from Trinity scripts that leverage the R software. In this context, 3792 and 3064 DE transcripts (of 49,162 and 54,194 total transcripts, respectively) were identified in “Leucocarpa” and “Cassanese.” The fold change and the statistical significance values between different developmental stage and cultivar were also estimated.

Trinity facilitates analysis of RNA-seq data, including scripts for extracting transcripts that are above some statistical significance (FDR threshold) and fold-change in expression. To examine expression across multiple samples, the FPKM expression values across samples have been normalize, which will account for differences in RNA composition, afterwards TMM normalization generate a matrix of normalized FPKM values across all samples.

**Figure 1:**
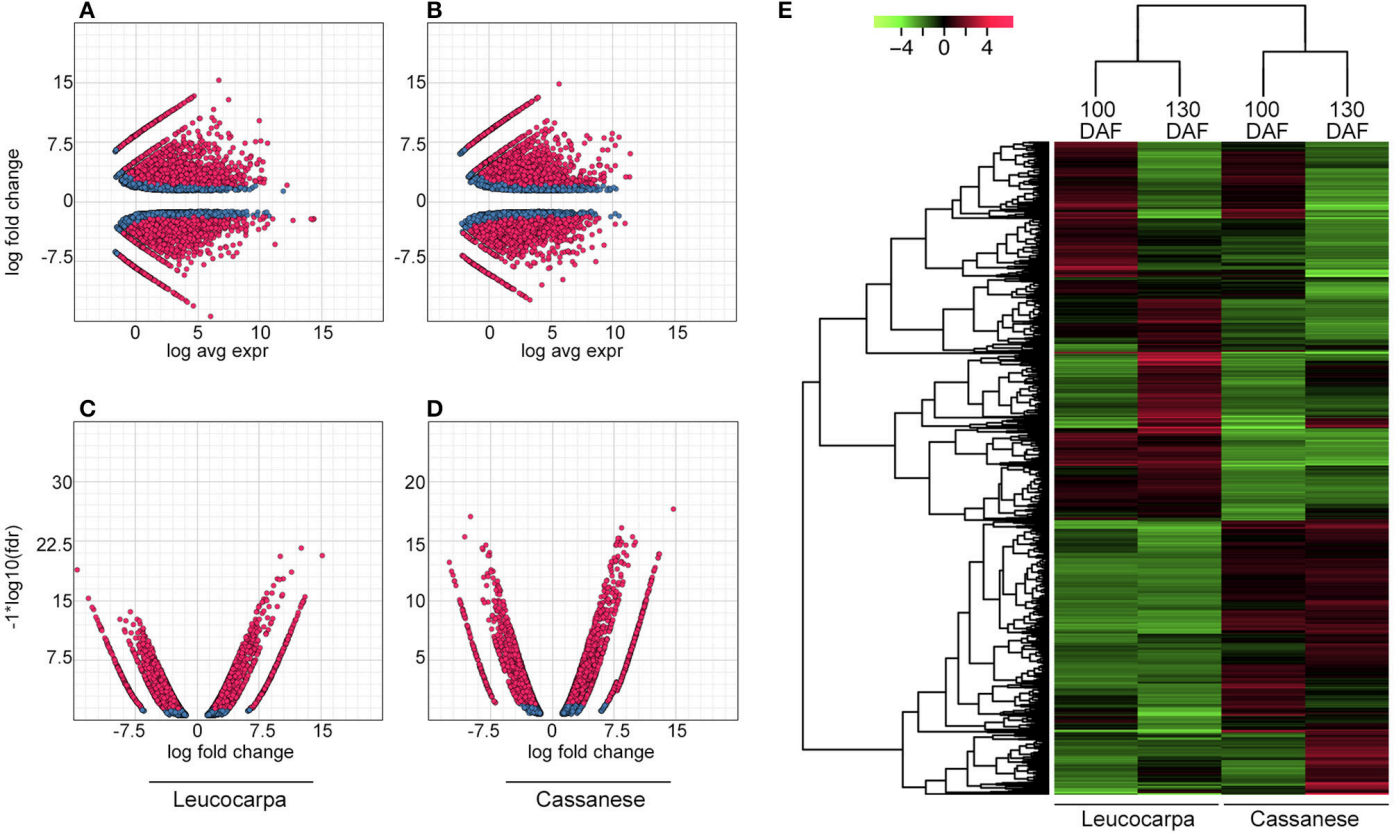
**Whole transcripts expression during fruit ripening**. In **(A,B)**, MA plot for differential expression analysis generated by EdgeR: for each gene, the log_2_ (fold change) (log_2_(100DAF/130DAF)) between the 100 and 130 DAF samples is plotted (A, y axis) against the gene's log_2_(average expression) (M, x axis). In **(C,D)**, the Volcano plot reports a FDR (−log _10_FDR, y axis) as a function of log_2_ (fold change) between the 100 and 130 DAF samples (logFC, x axis). Transcripts that are identified as significantly differentially expressed at most 0.1% FDR are colored in red. In **(E)**, the heat map show the relative transcript/sample expression levels. Green and red colors are used to indicate the transcripts up to four-fold up- and down- regulated, respectively. Expression values (FPKM) are log_2_ transformed and then median-centered by transcript. The dendrogram, on the left, orders whole transcripts set in relation to their level of expression.

These adjusted library sizes are used to recompute the FPKM expression values. Although the raw fragment counts are used for differential expression analysis, the normalized FPKM values are used below in examining profiles of expression across different samples, each DE set of transcripts was displayed as MA plots (where M = log ratios and A = mean values) (Figures 1A,B), volcano plots (Figures 1C,D) and clustered heat maps (Figure 1E). A correlation matrix (Figure S2) for the different developmental stages across cultivars, reveals that samples are more highly correlated within cultivar than between cultivar.

### Functional annotation of differentially expressed transcript sets

**Figure 2:**
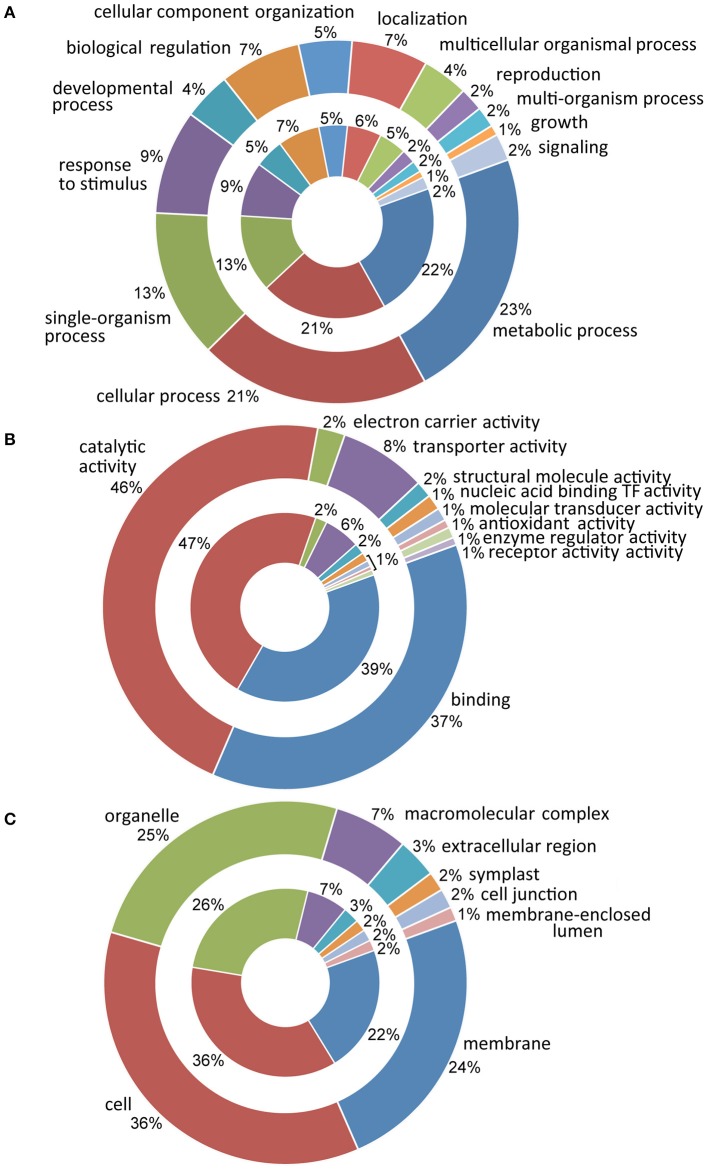
**Distribution of ontological categories (level 2 GO terms) in Leucocarpa (inner chart) and Cassanese cvs (outer chart) DE transcripts according to: biological process (A), molecular function (B) and cellular component (C)**. In A metabolic process and cellular process are the most represented groupings; the divisions relating to catalytic activity and binding are strongly represented in **(B)**, while in C cell, organelle and membrane categories are represented. At the side of anthological categories, the percentage of the transcripts within each class is reported.

The *in silico* analysis of the entire sets of DE transcripts, performed by querying databases of genes and proteins (NCBI, ExPASy, InterProScan) and the functional annotation software Blast2GO, have allowed for each sequence to be traced back to the gene family and to be annotated according to the terms of the three main Gene Ontology vocabularies (Figure 2). Since analyses were conducted on the same organ and developmental stages, in both analyzed cultivars a fairly overlapped distribution of GO terms was observed during the developmental transition. In particular, the most represented ontological categories were membrane (GO:0016020), cell (GO:0005623) and organelle (GO:0043226). Molecular functional categories were strongly represented by terms related to catalytic activity (GO:0003824) with 47 and 46% in Leucocarpa and Cassanese cvs, respectively, followed by binding (GO:0005488) and transporter activity (GO:0005215). Finally, more than 10 categories were identified at the biological process level with metabolic and cellular processes (GO:0008152, GO:0009987), among the groups most represented, highlighting the intense and complex metabolic and regulatory activities during fruit maturation.

In order to trace back to the pathways, such as flavonoids and anthocyanin, (map 00941 and 00942, Figures S3A,B, respectively), which were more closely involved in the transition between 100 and 130 DAF, the whole DE transcripts set was examined through the Kyoto Encyclopedia of Genes and Genomes (KEGG). Functional analysis was implemented in Mapman, to focus gene expression changes via Image Annotator. All obtained results are consistent with a down regulation of flavonoid and anthocyanins metabolism in Leucocarpa cv, while an opposite trend was observed in “Cassanese” (Figure S4).

### Gene expression during olive fruits ripening

We performed a quantitative RNA-seq analisys in a cultivar of *Olea europaea* species, whose fruits are characterized by a switch-off in skin color at full ripeness, to identify the genes involved in flavonoid andanthocyanin biosynthesis.

The transcripts set in flavonoid and anthocyanin pathways were identified in our Illumina datasets. It includes 11 transcripts: phenylalanine ammonia lyase (PAL), cinnamate 4-hydroxylase (C4H), 4-coumarate-CoA Ligase (4CL), chalcone synthase (CHS), chalcone isomerase (CHI), flavanone 3-hydroxylase (F3H), flavonol 3′-hydrogenase (F3′H), flavonol 3′5 ′-hydrogenase (F3′5′H), flavonol synthase (FLS), dihydroflavonol 4-reductase (DFR), anthocyanidin synthase (ANS), UDP-glucose: anthocianidin:flavonoid glucosyltransferase (UFGT) (Supplementary data, Table S1). Moreover, it was possible to identify different member of MYB, MYC and WD transcription factors related to the regulatory complex that controls anthocyanin structural genes at the transcriptional level (Takahama, 2004).

**Figure 3:**
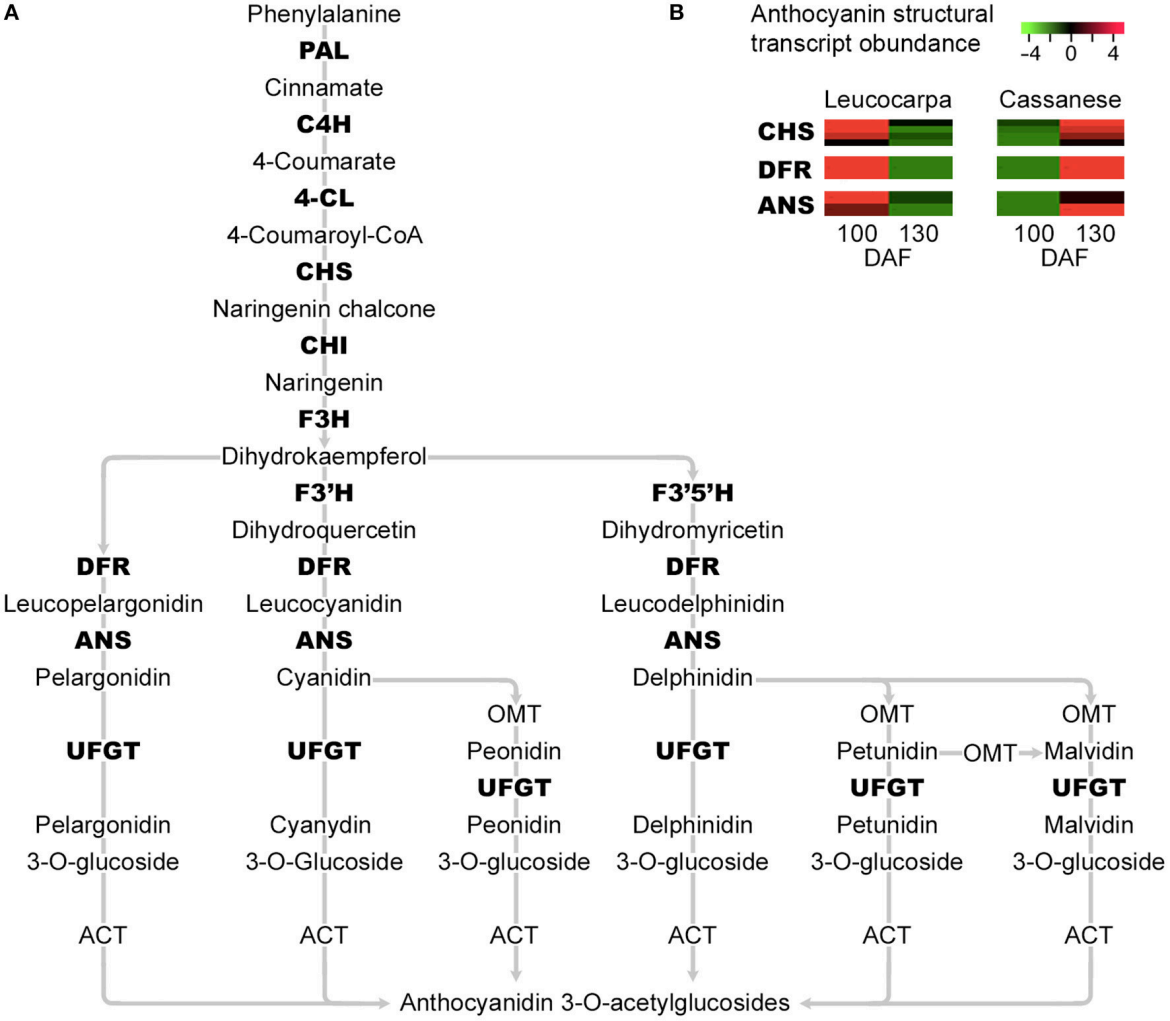
**Simplified representation of the main steps in the flavonoid and anthocyanin enzymatic pathways**. The transcripts identified as differentially expressed in the 100–130 DAF transition and which show a reduced expression in the “Leucocarpa” epi-mesocarp at 130 DAF and an opposite expression pattern in “Cassanese” epi-mesocarp, that leads at 130 DAF to a normal veraison stage are indicated in bold (A). *Phenylalanine ammonia lyase* (*PAL*), *cinnamate 4-hydroxylase* (*C4H*), *4-coumarate-CoA Ligase* (*4CL*), *chalcone synthase* (*CHS*), *chalcone isomerase* (*CHI*), *Flavonol 3-hydrogenase* (*F3H*), *Flavonol 3*′*-hydrogenase* (*F3*′*H*), *Flavonol 3*′*5* ′*-hydrogenase* (*F3*′*5*′*H*), *dihydroflavonol 4-reductase* (*DFR*), *anthocyanidin synthase* (*ANS*), and *UDP-glucose: anthocianidin:flavonoid glucosyltransferase* (UFGT). In **(B)**, the expression abundance of anthocyanin structural genes *CHS, DFR*, and *ANS* identified in our whole transcript expressions analysis are highlighted. Each row show the relative expression abundance of transcript clusters; green and red colors are used to indicate the transcript levels four-fold up- and down- regulated, respectively.

Interestingly, the quantitative gene expression analysis does not seem to show significant differences during olive fruit development in Leucocarpa and Cassanese cvs (Table 2). Indeed, focusing attention on the paths that control the biosynthesis of pigments and the natural reduction of photosynthetic pigments during the veraison stage (Pua and Davey, 2010), the “Leucocarpa” was characterized by a broad down-regulation of CHS, DFR, and ANS transcripts (Figure 3), during the 100–130 DAF transition compared to Cassanese cv.

**Table 2:** **Number of differential expressed transcripts during 100–130 DAF transition for each cultivar**.

**Sample**	**Total transcripts**	**DE trascripts**
Leucocarpa 100–130 DAF	49,162	3792
Cassanese 100–130 DAF	54,194	3064

**Figure 4:**
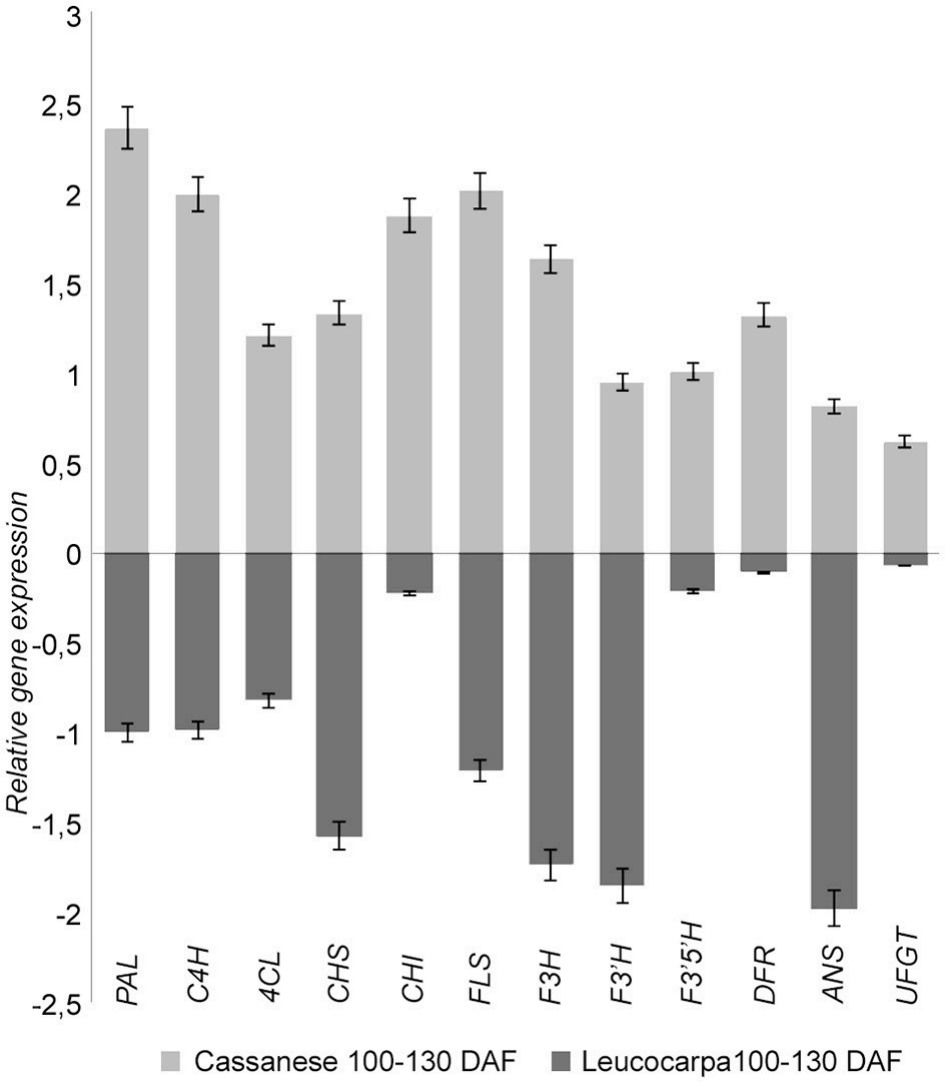
**Relative transcripts expression during fruit ripening in Leucocarpa (dark gray) and Cassanese (light gray) cvs**. The qRT-PCR results (log fold change) are presented as a proportion of the highest value after normalization with the EF1 house-keeping gene; for each cv 100 DAF samples are used as calibrator. The means ± s.e. of two independent biological replicates are reported.

The estimated fold change of the selected genes was also confirmed by quantitative PCR experiments (Figure 4). In particular, the expression of transcripts putatively involved in the selected pathway were more highly expressed in “Cassanese” genotype than in “Leucocarpa.”

**Figure 5:**
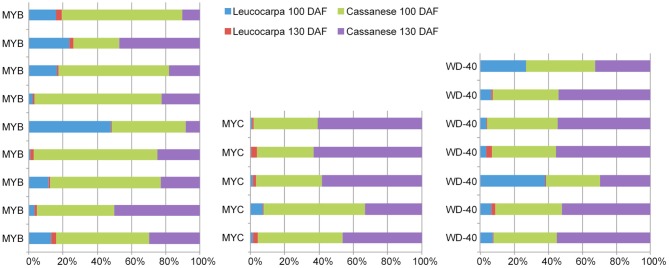
**Comparisons in ***MYB***, ***MYC***, and ***WDR*** transcripts abundance between samples**. Each data is displayed as a stacked bar. Transcripts expression levels were taken from the complete FPKM normalized matrix that were identified as differentially expressed.

This genome-wide overview on flavonoid and antocyanidin genes also allowed us to select different members of MYB, MYC, and WD transcription factors (TF), within the differentially expressed gene set, linkable to anthocyanins regulatory circuit (Dixon et al., 2005; He et al., 2008; Tian et al., 2008; Stommel et al., 2009; Jaakola, 2013). The abundance estimation analysis made it possible to compare the identified TFs in all analyzed samples. In the “Cassanese” plant, despite a slight decline, the amount of transcripts during 100–130 DAF transition was consistent with the increased anthocyanin structural gene expressions and metabolite accumulation during growth of fruits; whereas in the Leucocarpa cv the identified TFs are primarily characterized by lower expression levels and a general reduction in expression abundance during ripening transition. The differences were most evident when the comparison was carried out at the same stage (100 or 130 DAF) of maturation. 9 MYB, 5 MYC, and 7 WD TF undergo a decrease in expression during transition, in contrast to Cassanese cv where they appear to participate in the activation pathway (Figure 5).

## Discussions

In the present work we used the Illumina RNA-seq technology to identify the transcripts along flavonoids and anthocyanins biosynthetic pathways and to monitor their expression levels during ripening, by comparing two olive cultivars characterized by different phenological behavior at ripening in terms of anthocyanin accumulation and general pigmentation. We also used a *de novo* transcriptome assembly strategies performed in many plants, including rice, maize, sesame, bamboo, poplar, sweet potato, *Eucalyptus* tree, chickpea, and orchid (Mizrachi et al., 2010; Wang et al., 2010; Fu et al., 2011; Garg et al., 2011; Wei et al., 2011; Zhang et al., 2011a).

The characterization of the genetic entity of olive cultivars has benefited from new molecular biology and high-throughput sequencing methods (Alagna et al., 2009; Galla et al., 2009; Bazakos et al., 2012; Muñoz-Mérida et al., 2013). Through the analysis of massive data it is possible to identify/investigate the genetic pathways that underlie specific, or more general, agronomic traits in the physiological performance of the plants belonging to the *Olea europaea* species.

Between different high-throughput methods, the Illumina sequencing is the best next generation technology, both less costly and more efficient, for transcriptome analysis, if compared with 454 platform, in particular when used in non-model organisms, where genomic sequences are unknown.

Even though this technology has been previously restricted to the re-sequencing of organisms with available reference genomes (Nagalakshmi et al., 2008), its recent improvement has enabled the development of *de novo* strategies for robust trascriptome reconstruction for non-model plants from short reads and their assembly into unigenes.

Through this approach we identified anthocyanin genes, including PAL, C4H, 4CL, CHS, CHI, F3H, F3′H, F3′5′H, FLS, DFR, ANS, from two olive cultivars.

In addition, different transcription factor members with similarity to MYB, MYC, and WD40 family and involved in anthocyanin biosynthesis were also found. Furthermore, the transcripts abundance of identified genes was correlated to the accumulation rate of anthocyanin metabolites.

The anthocyanin biosynthesis pathway has been extensively studied in several plant species, such as petunia, pears, goji berry, bilberry and black raspberry (Jaakola et al., 2002; Zeng et al., 2014). During the ripening progression, many species including the olive tree accumulate anthocyanin in their fruits (Jaakola et al., 2002; Sweetman et al., 2009; Zhang et al., 2011b). In this context, anthocyanins are considered potent marker to monitor ripening stages and organoleptic quality of fruits.

In apple, the regulatory circuit in anthocyanin biosynthesis is tuned by the MYB-MYC-WD40 protein complexes (Ramsay and Glover, 2005; Schaart et al., 2012). Moreover the R2R3-MYB and bHLH TFs are able to activate structural genes, including CHS, DFR and ANS, and ultimately promote anthocyanin accumulation in fruits (Chagné et al., 2013; Umemura et al., 2013; Zeng et al., 2014). In our case the transcripts abundance of MYB, MYC, and WD40-*type* TFs was higher in Cassanese cultivar than in Leucocarpa and was also directly related to anthocyanin accumulation.

In conclusion, the comparative approach performed provide an invaluable resource to identify genes involved in fruit maturation and to define the metabolic pathway and tissue specific functional genomics in non-model plant species. The characterization of transcripts from flavonoid and anthocyanin biosynthetic pathways and the analysis of their expression level in olive fruits is an important goal to understand the veraison event of fruits and to increase the knowledge on these antioxidant molecules, important for human health.

## Author contributions

DI performed research and discussed results. AC designed research analyzed data and discussed result. IM designed research analyzed data and discussed results. All authors contributed to improving the papers and approved the final manuscript.

### Conflict of interest statement

The authors declare that the research was conducted in the absence of any commercial or financial relationships that could be construed as a potential conflict of interest.

## Footnotes

^1^FAOSTAT 2008 home page Columbia URL: http://www.columbia.edu/cgi-bin/cul/resolve?ASL9609

## Supplementary material

Figure S1 **“Cassanese” (A–A″) and “Leucocarpa” (B–B″) fruits, in the top and bottom respectively, sampled during 100 (A,B) and 130 (A′,A″,B′,B″) DAF transition**.

Figure S2 **Correlation matrix for sample across cultivar: samples are more highly correlated within cultivar than between cultivar**.

Figure S3 **KEGG pathway for flavonoid and anthocyanins biosynthesis, map: 00941 (A) and 00942 (B)**. The isoforms of differentially expressed transcripts controlling flavonoid as well as anthocyanin biosynthesis were mapped.

Figure S4 **MapMan visualization of changes in expression levels of genes associated with secondary metabolism**. Green denotes down-regulation and red up-regulation. Changes in **(A,B)** gene expression after 130 DAF compared to 100 DAF as calibrator, in Cassanese and Leucocarpa cvs respectively.

Table S1 **Sequences list and qRT-PCR primers set to validate selected targets**.
