# Supplementary figures and images for: A De novo Transcriptomic Approach to Identify Flavonoids and Anthocyanins “Switch-Off” in Olive (Olea europaea L.) Drupes at Different Stages of Maturation
Source: Front Plant Sci. 2016 Jan 19;6:1246. doi: 10.3389/fpls.2015.01246 (PMC4717290; doi:10.3389/fpls.2015.01246)

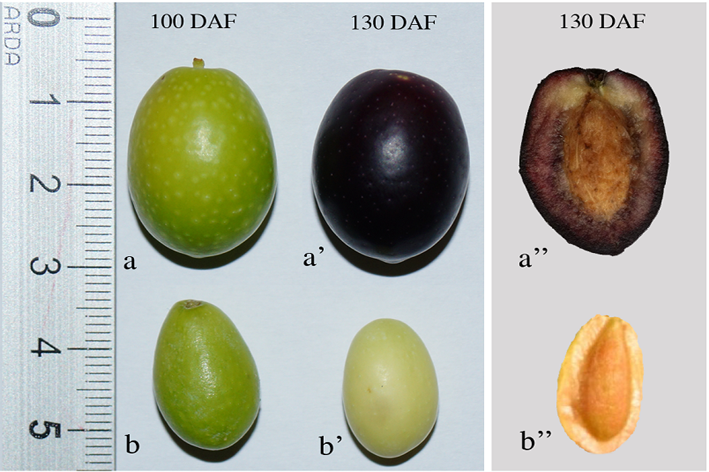

Supplement: Figure S1 — “Cassanese” (A–A″) and “Leucocarpa” (B–B″) fruits, in the top and bottom respectively, sampled during 100 (A,B) and 130 (A′,A″,B′,B″) DAF transition. [file Image1.TIF]

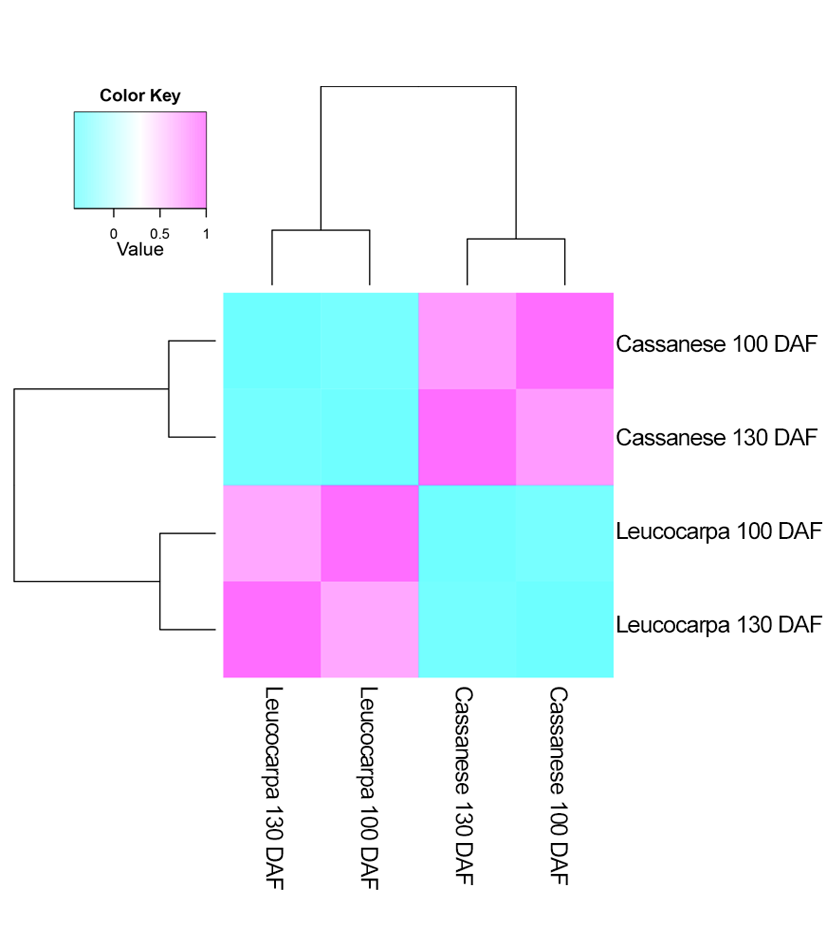

Supplement: Figure S2 — Correlation matrix for sample across cultivar: samples are more highly correlated within cultivar than between cultivar. [file Image2.TIF]

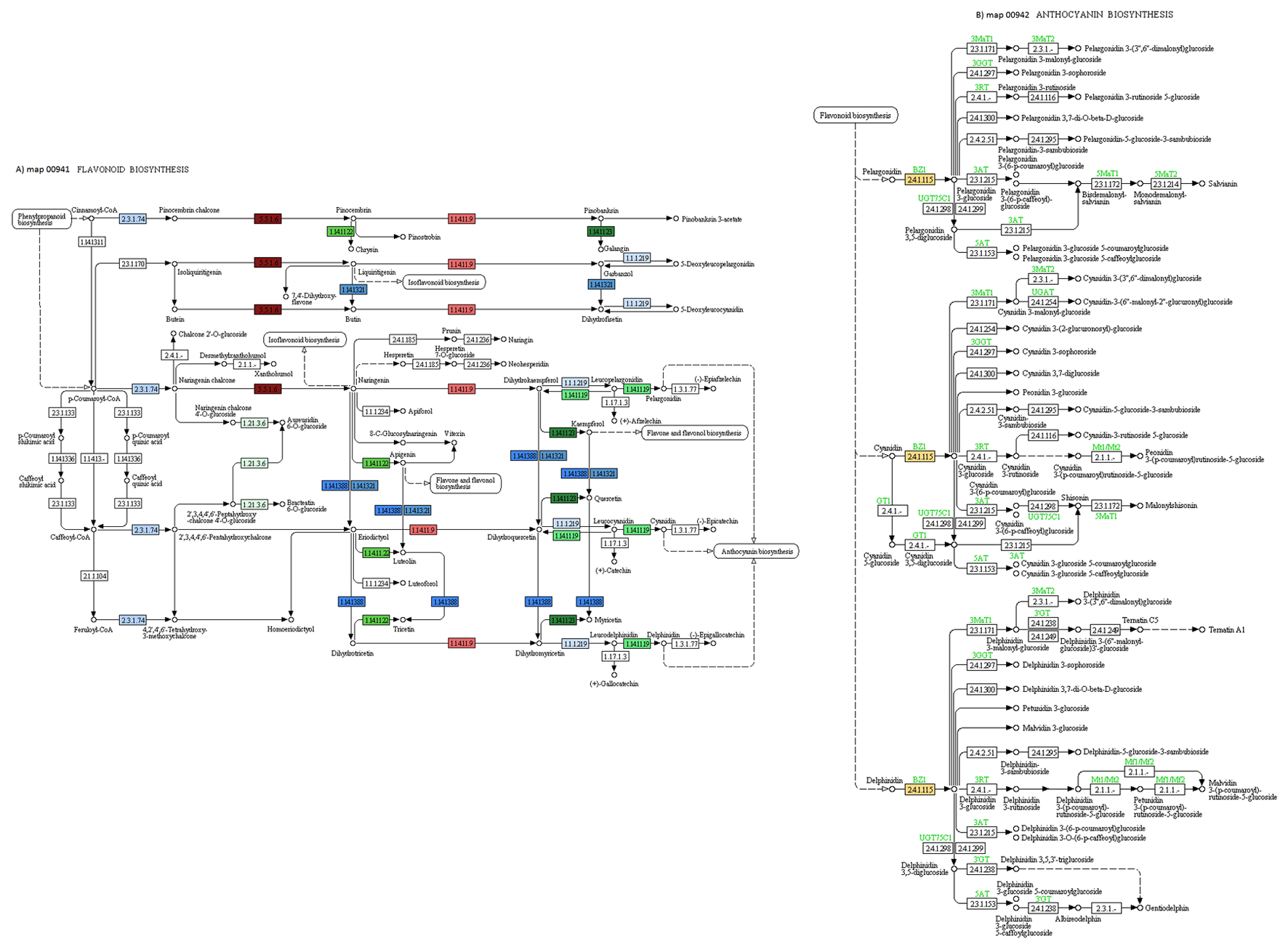

Supplement: Figure S3 — KEGG pathway for flavonoid and anthocyanins biosynthesis, map: 00941 (A) and 00942 (B). The isoforms of differentially expressed transcripts controlling flavonoid as well as anthocyanin biosynthesis were mapped. [file Image3.TIF]

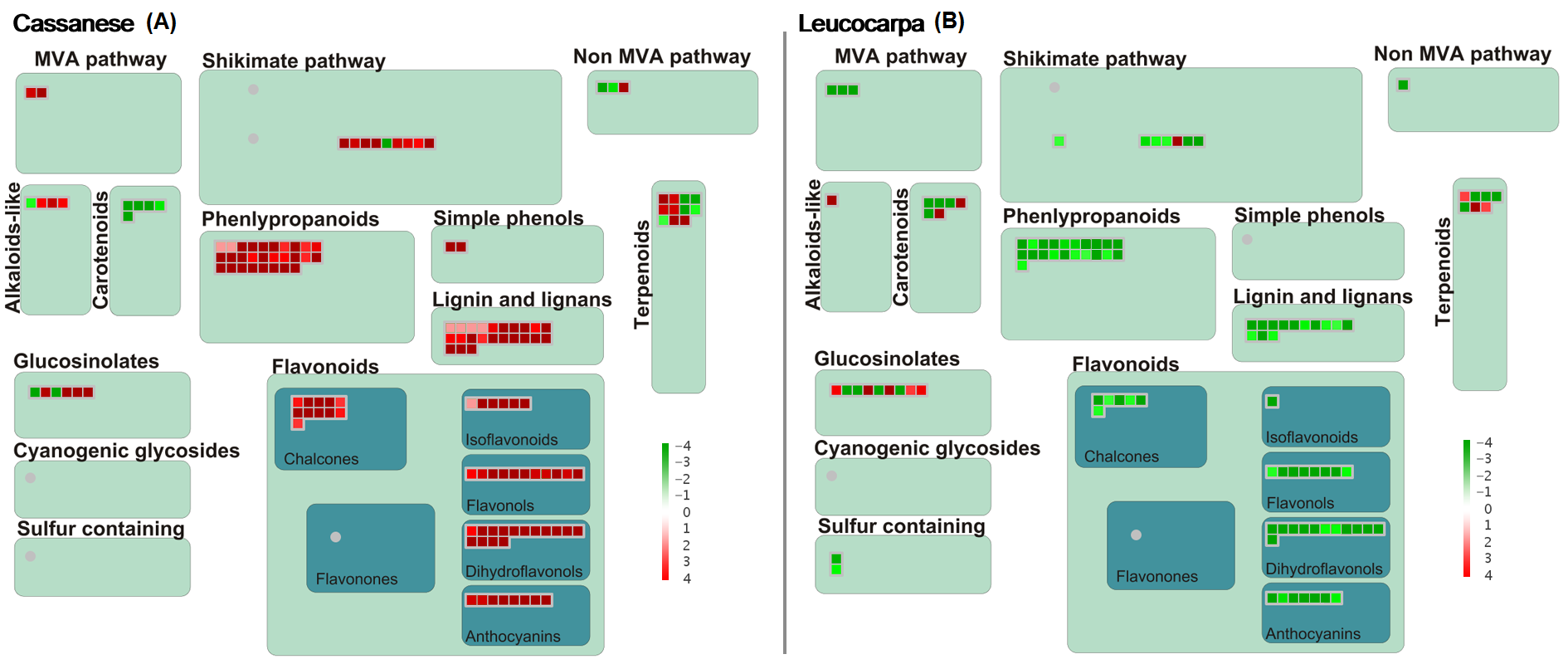

Supplement: Figure S4 — MapMan visualization of changes in expression levels of genes associated with secondary metabolism. Green denotes down-regulation and red up-regulation. Changes in (A,B) gene expression after 130 DAF compared to 100 DAF as calibrator, in Cassanese and Leucocarpa cvs respectively. [file Image4.TIF]
