# Supplementary material for: A De novo Transcriptomic Approach to Identify Flavonoids and Anthocyanins “Switch-Off” in Olive (Olea europaea L.) Drupes at Different Stages of Maturation
Source: Front Plant Sci. 2016 Jan 19;6:1246. doi: 10.3389/fpls.2015.01246 (PMC4717290; doi:10.3389/fpls.2015.01246)
Supplement: Table S1 — Sequences list and qRT-PCR primers set to validate selected targets. [file Table1.pdf]

Table S1.

Transcript	Size (bp)	Primer FW	Primer BW	Amplicon (bp)
PAL	2466	5'-ACACATCCATCTTCCAAAAG-3'	5'-GTTCCCAGTTCTTCCCTTAC-3'	175
C4H	311	5'-CTCCACCATTGTCTTAAAGC-3'	5'-TCCATACATGCAATAAACCA-3'	150
4CL	1587	5'-AAATTTAAAGGCTTCCAGGT-3'	5'-GCTTCTTCGGTAAGTTCAAA-3'	164
CHS	826	5'-GATTGGAACTCGATTTTCTG-3'	5'-GGACTTTCTCATCTCATCCA-3'	177
CHI	1354	5'-AGGGTTCACGTATGGAGTG-3'	5'-TGCAAATACAATCTCAGCAG-3'	194
FLS	1313	5'-GAATAGAGTCACTGTCAAGGG-3'	5'-TCCCTAACCATCGTGGCCTTT-3'	168
F3H	1055	5'-TCCTCTGCCCGTGTGATAGT-3'	5'-AATCCGTGTGATGCAGTGAG-3'	226
F3'H	995	5'-GTGGCAGAAGCTGACCTACC-3'	5'-CGTAGAGCCCTTTGGAATGA-3'	150
F3'5'H	1971	5'-AGTGGTCACCAATGGGATGT-3'	5'-CACATCAAACGTGGCTCATT-3'	160
DFR	1631	5'-ATTTCAGGTGTTGGCTGAGG-3'	5'-ATTCCATATGGCCAGGTCAA-3'	156
ANS	1133	5'-GCATAGGGTCACTGTCAATGG-3'	5'-TCCTTACCATCATGGCCTTT-3'	186
UFGT	460	5'-AATGGCTTTGATGGAAGGTG-3'	5'-TTCACGCTGGCATAAACTCA-3'	166
